# Experiment *in vivo*: How COVID-19 Lifestyle Modifications Affect Migraine

**DOI:** 10.3389/fneur.2021.744796

**Published:** 2021-10-05

**Authors:** Vesselina Grozeva, Ane Mínguez-Olaondo, Marta Vila-Pueyo

**Affiliations:** ^1^Neurology Practice Polyclinic, Sofia, Bulgaria; ^2^Department of Neurology, Hospital Universitario Donostia, San Sebastián, Spain; ^3^Headache Group, Wolfson Centre for Age Related Diseases, Institute of Psychiatry, Psychology and Neuroscience, King's College London, London, United Kingdom

**Keywords:** COVID-19, pandemic, migraine, lifestyle, modifications

## Abstract

**Introduction:** The coronavirus disease 2019 (COVID-19) pandemic represents a unified lifestyle modification model, which was developed by the globally applied measures. The lockdowns designed the perfect study settings for observing the interaction between migraine and the adopted changes in lifestyle. An experiment *in vivo* took place unexpectedly to determine how the lockdown lifestyle modifications can influence migraine.

**Subsection 1: Overall lifestyle modifications during the pandemic:** People stay home, and outdoor activities and public contacts are restricted. Sleep is disturbed. Media exposure and prolonged screen use are increased. Working conditions change. In-person consultations and therapies are canceled. The beneficial effects of short-term stress, together with the harmful effects of chronic stress, were observed during the pandemic.

**Subsection 2: Short-term effects:** Substantial lifestyle changes happened, and knowing how vulnerable migraine patients are, one could hypothesize that this would have resulted in severe worsening of headache. Surprisingly, even though the impacts of changing social conditions were significant, some patients (including children) experienced a reduction in their migraine during the first lockdown.

**Subsection 3: Long-term effects:** Unfortunately, headache frequency returned to the basal state during the second pandemic wave. The risk factors that could have led to this worsening are the long-term disruption of sleep and dietary habits, stress, anxiety, depression, non-compliance to treatment, and working during the pandemic.

**Discussion:** Sudden short-term lifestyle changes taking migraine patients out of their usual routine may be beneficial for headache management. It is not necessary to have a natural disaster in place for a drastic lifestyle modification with 6–8-week duration, if we know that this will improve migraine.

## Introduction

Migraine is an invisible disabling and undertreated disease (1). It is among the most prevalent disorders and one of the main causes of disability worldwide (2). A relationship between migraine and some environmental factors exists (3). However, the exact pathophysiology and how such factors affect the course of migraine remain unclear (4). Among the described triggers and aggravating factors (5) are emotional stress (6), sleep disturbances (7), nutrition, physical exercise, and others (8, 9). Avoidance of these triggers/factors through lifestyle changes is recommended because there is evidence that certain modifications of lifestyle can be beneficial for a better management of migraine (5, 6, 8). Despite the lack of well-designed studies to prove this link, the coronavirus disease 2019 (COVID-19) pandemic represents a unified lifestyle modification model that was followed in several countries because the preventative measures were globally applied. The COVID-19 lockdown designed the perfect study settings for observing the interaction between migraine sufferers and the adopted changes in lifestyle. This exceptional *in vivo* experiment took place unexpectedly and allowed us to determine how the lockdown lifestyle modifications can influence migraine.

## Subsection 1: Overall Lifestyle Modifications During the Pandemic

Substantial lifestyle changes happen during pandemic lockdowns, especially when global restrictions are applied. Social isolation is required to prevent spreading of the disease. People stay home, and outdoor activities and public contacts are restricted (9). Working conditions and some jobs change, while others disappear completely (10). Home-based working combined with homeschooling children under one and the same shelter carries additional burden. Parent–child relationships become challenging when all family members are required to stay close together under one shelter for a long time. Caregiving fatigue can add on (11, 12). Maintaining regular eating habits during an outbreak can become a real challenge as well (11). Shopping food restrictions and related food insecurity have been linked to risk of weight gain and obesity (13). Weight gain is very likely to happen during lockdown. Closed restaurants and limited physical exercise and outdoor activities could facilitate it (14). Of note, obesity is negatively associated with increased migraine frequency and disability (15). Both weight gain and obesity can be accompanied by sedentary life features such as prolonged sitting and poor posture, which are also very common in lockdowns and can be triggers of migraine attacks (16, 17). On the same line, the reduction in physical activity as a direct effect of lockdowns can only be a negative factor for migraine frequency, intensity, and duration (11). Sleep is another factor that could be possibly disturbed when having to cope with the pandemic emergency, increased stress, and fear of the unknown. Such disturbances can affect mood and quality of life (18), and this is essential for all aspects of health (11). Poor sleep has been linked to headache severity and disability, especially in the youth (19, 20). Media exposure, constant influx of news, and prolonged screen use can lead to shorter and poorer quality of sleep (21); during the pandemic, a higher social media exposure was associated with severe psychological distress among migraineurs (22, 23) and also with an increased risk of migraine in adolescents and young adults (24). Besides the increased use of screens, brightness of computer screens is also considered a trigger for migraine attack exacerbation (25). Furthermore, it is important to mention that usual healthcare is modified during a pandemic, and in-person consultations and migraine therapies are canceled or substituted (10). It is well-known that pandemics increase stress, anxiety, and negative emotional reactions such as anger, fear, and risk perception (26). Social isolation, in turn, produces depressive symptoms, uncertainty about the future, and concerns for the health of family members (27).

The COVID-19 pandemic is a stressor, and its effects on migraine patients can be divided into short and long term. Short-term stress is known to activate multiple physiological systems as a response to enable survival (28) and may improve adaptive mechanisms. However, a transition can be observed from adaptive short-term stress to harmful chronic stress (28). Chronic stress results in stress-related biological changes that can last for months and even years (28). It may be hypothesized that under certain conditions, chronic prolonged stress could worsen a disease (28). The COVID-19 pandemic can be considered as an ideal experiment to observe the beneficial effects of short-term stress (during the beginning of the pandemic and the first lockdown), together with the harmful effects of the chronic stress observed during the ongoing pandemic. The COVID-19 pandemic has modified daily life in a negative way, and knowing how vulnerable migraine patients are, it could be suggested that this natural disaster would have resulted in severe worsening of the disease. Surprisingly, the study data show different results, especially for the first lockdown that could be possibly related to the short-term effects of the changes that occurred in the beginning of the pandemic.

## Subsection 2: Short-term Effects

The COVID-19 pandemic entirely changed the daily living of migraine patients. Contrary to the expectations, several studies have shown that the lockdown has been beneficial for different groups of migraine patients. A study conducted in Italy showed that the lockdown led to a reduction in reported headache intensity and frequency in Italian children and adolescents (29). Another study investigated the impact of COVID-19 on young migraine patients. The results of the survey of the study showed a reduction in reported headache intensity and frequency as well (10, 29). A subsequent Dutch study also proved that the change in lifestyle caused by the pandemic has been beneficial for individuals with migraine (14). The number of migraine days and the acute medication intake days decreased along the first lockdown (14). During that period, an increase in general well-being was noted, compared with the month before the lockdown. Furthermore, for those patients who provided extended electronic diary data, this change was maintained even during the 2nd month of lockdown (10, 14). A Japanese group initially hypothesized that increased stress during the COVID-19 pandemic would affect the migraine population in a negative way (30). In line with this, a significant number of patients reported increased stress, and a negative impact of the first wave of the COVID-19 pandemic on their daily lives, together with concerns about COVID-19 and changes in mood and sleep. However, headache-related disability, the number of headache days and headache intensity did not change during the first wave of the COVID-19 pandemic. Thus, the results did not support the hypothesis for a negative impact on migraine course during the first lockdown (30). This study, though, highlighted the development of a new-onset headache in younger age individuals with worsened mood, sleep, and increased stress due to the pandemic (30).

## Subsection 3: Long-term Effects

The COVID-19 lockdowns have led to multiple lifestyle changes, which turned out to be time and quantity dependent in terms of effects on migraine. Long-lasting insecurity and stress related to the pandemic could potentially have led to an increased migraine disability (14). In contrast with the evidence presented in the previous section, a Spanish study found out that the overall long-term effects of COVID-19 lockdowns on migraine are negative (9). Approximately half of the studied patients shared a negative lockdown-related impact on their headache (9). The worsening was represented by higher intensity of attacks, and it was associated with higher levels of post-traumatic stress, anxiety, and depression (9). Increased intensity and frequency of migraine during lockdown were both related to new, but long-term triggers, such as changes in life habits or insomnia, and the negative emotional impact of COVID-19 (9). This phenomenon was explained with moderate-to-severe symptoms of post-traumatic-stress (9). Psychological distress among migraine patients is considered as one of the most important factors for worsening and poor management of migraine (31). Another study conducted in July 2020 during lockdown showed increased migraine frequency and severity among the majority of patients with migraine in Kuwait. Migraineurs were overusing analgesics, and around 10% transformed to chronic migraine during lockdown (31). Sixty percent reported worsening of their headaches, and 80 percent reported anxiety and depression. The risk factors that led to this worsening were sleep disturbances and bad dietary habits, associated with anxiety and depression, lack of communication with their treating neurologist, non-compliance to treatment, and working during the pandemic (31). Chinese patients with migraine also reported increased levels of insomnia, anxiety, and depressive symptoms during the COVID-19 pandemic (32). Perceived stress was more strongly associated with brooding and COVID-related rumination among patients with migraine than healthy controls (33). Perceived stress is known to be more common in chronic migraine, depression, and anxiety (34) and it might have been one of the risk factors that contributed to the worsening of migraine during the pandemic. The positive effects on migraine from the first lockdown in Italy reverted to negative during the second lockdown, especially for the patients with episodic migraine (26). This resulted from more pronounced emotional reactions (anger, disgust, fear) toward the pandemic in the second wave that were correlated to higher headache intensity and frequency (26). Negative psychological effects and stressors included longer quarantine duration, factors associated with infection, economic crisis, and uncertainty of government measures (35). Frequency of migraine came back to its basal condition, and even worsened in episodic patients, with a potential transformation into chronic form (26). Thus, the long-lasting effects of the pandemic were found to have a negative impact on migraine evolution (33).

## Discussion

The COVID-19 pandemic lockdowns entirely changed the daily living of migraine patients. Multiple lifestyle changes modified the susceptibility to a migraine attack (14). Professionals initially suggested that migraine patients should stick to their previous routine to avoid worsening of their headache during the pandemic (31). However, our clinical practice and studies showed that the sudden and abrupt change in their routine was initially favorable for migraine patients. Stressful, challenging, and emotionally overwhelming experiences in the school environment may cause overreaction of the central nervous system to environmental requests that are perceived as too intense by the individual. Thus, the risk of headache and migraine is increased (36). This is also valid for the adult population, although the environment is different, and this population is considered to have more resilience to deal with such stressors. Lifestyle modifications during lockdown seem to have led to a reduction in the intensity and frequency of migraine in children and adolescents (29). These effects were explained mainly with the lower level of school-related stress during lockdown (29). We agree with the speculation that these unexpected improvements in migraine symptoms could be related to a reduction in external or internal demands for high performance in daily social settings, such as school/or work and sport or leisure activities (37, 38). Psychological stressors are potential migraine triggers. In the beginning of this unknown natural disaster, it was difficult to determine which were the bigger stressors: the usual daily demands that might had exceeded the capability of migraine patients or the fear of the unknown virus that took over the whole world, but there was still little information about it. A logical explanation for the improvement of migraine during the first pandemic wave could be the reduction of all known stressors in daily life. Since the actual hazard and harm of COVID-19 disease could not have been estimated in the beginning of the pandemic, the elimination of the old and usual stressors seems to have been very beneficial. The Dutch group proposed that working flexibility, reduction in social demands, and freedom to choose how to organize one's time might have led to a reduction in migraine frequency, intensity, and disability (14). This speculative conclusion was made for both children and adults with migraine (14, 29). The appropriate modification of the impact of school, work, and social demands on migraine patients could be beneficial for their migraine symptoms, and actually, the results indicate that short-term changes in some elements of their lifestyle can have a positive influence on the course of migraine (10).

In the first pandemic wave, the limited impact of the emotional behavior on migraine may be explained by the occurrence of a phenomenon called resilience (26). Unfortunately, during the second wave, negative psychological effects due to the prolonged stress and health emergency state prevailed (35). Negative emotional reactions against the pandemic, such as anger, anxiety, fear, and risk perception, linked to headache intensity, were absent during the first lockdown (26). The second wave of the pandemic led to an extension of the restrictive measures (35) and the emotional impact of the quarantine was enhanced by the uncertainty about the pandemic outcome, restrictive measures, and continuous viral diffusion (35). In the long term, factors like insecurity and stress may have worsened migraine disability (14). This COVID-19 experiment *in vivo* taught us that sudden short-term lifestyle changes that take migraine patients out of their usual routines may be beneficial for headache management. It is not necessary to have a natural disaster in place for a drastic lifestyle modification with 6–8-week duration, if we know that this will improve migraine. Short-lasting abrupt changes of lifestyle under less stressful circumstances can have even larger benefits than those observed in the first COVID-19 lockdown (14).

## Limitations

This review provides a narrative approach by using all the current evidence to show the positive impact of the first COVID-19 wave on migraine patients. We acknowledge that using this approach, and not a systematic review, could be considered a limitation of the study.

We did not compare the effects of COVID-19 pandemic in migraine patients with previous pandemics because of several reasons. First, previous pandemics, such as the one in 1918, happened in a different historical moments where lifestyle was completely different from the current one, making the comparison irrelevant. Another point is the global effect that the COVID-19 pandemic has had, which is a unique characteristic of this pandemic. Future research could investigate further what is common between the pandemic eras and what is clearly distinct in terms of lifestyle, technology, and medicine evolution.

It would be interesting to understand if the pandemic has also modified other diseases or if it is a unique feature of migraine. Although there are some studies that support the changes induced by the pandemic in patients with Parkinson's disease or multiple sclerosis (39, 40), it is difficult to establish a comparison with migraine as those studies do not make a distinction between the two pandemic waves.

## Data Availability Statement

The original contributions presented in the study are included in the article/supplementary material, further inquiries can be directed to the corresponding author.

## Author Contributions

VG created the perspective idea and drafted the first manuscript. AM-O, VG, and MV-P reviewed the literature, processed and analyzed the data, interpreted the results, and edited and reviewed the language. MV-P conceptualized and designed the article and critically revised the manuscript. All authors contributed to the article and approved the submitted version.

## Funding

MV-P received support from the Wellcome Trust (Grant number 104033). There were no funders involved in the study design, collection, analysis, interpretation of data, the writing of this article, or the decision to submit it for publication.

## Conflict of Interest

The authors declare that the research was conducted in the absence of any commercial or financial relationships that could be construed as a potential conflict of interest.

## Publisher's Note

All claims expressed in this article are solely those of the authors and do not necessarily represent those of their affiliated organizations, or those of the publisher, the editors and the reviewers. Any product that may be evaluated in this article, or claim that may be made by its manufacturer, is not guaranteed or endorsed by the publisher.
